# Supplementary material for: WordCommentsAnalyzer: A windows software tool for qualitative research
Source: F1000Res. 2018 Sep 4;7:536. Originally published 2018 May 3. [Version 2] doi: 10.12688/f1000research.14819.2 (PMC6137411; doi:10.12688/f1000research.14819.2)
Supplement: Supplementary file 3 [file f1000research-7-17614-s0002.tgz › b2a79496-4ada-47c4-9682-a99ce867836d.zip!/Eight Iranian Health Journals/Archives of Rehabilitation_26.docx]

© Archives of Rehabilitation. This work is licensed under CC BY-NC 4.0 (https://creativecommons.org/licenses/by-nc/4.0/).

Volume & Issue: دوره 13 - شماره 3

Title: بررسی تأثیر برنامه حمایتی سلامت‌محور بر کیفیت زندگی مادران کودکان فلج مغزی

http://rehabilitationj.uswr.ac.ir/article-1-974-fa.html

هدف: وجود کودک مبتلا به فلج مغزی کیفیت زندگی مادر را در تمام ابعاد متأثر می‌سازد. لذا پژوهش حاضر با هدف تعیین میزان تأثیر برنامه حمایتی سلامت‌محور بر کیفیت زندگی مادران کودکان فلج مغزی انجام شد. روش بررسی: مطالعه حاضر از نوع نیمه‌تجربی است که با انتخاب غیر‌تصادفی در دسترس دو گروه آزمون (30 نفر) و شاهد (30 نفر) انجام شد. برنامه حمایتی سلامت‌محور در سه مرحله بررسی و شناخت، حمایت و ارزشیابی اجرا شد. ابزارهای گرد‌‌آوری اطلاعات شامل پرسشنامه اطلاعات دموگرافیک و کیفیت زندگی Sf36 بود. داده‌ها قبل و دو ماه بعد از مداخله جمع آوری و توسط نرم افزار SPSS 16 مورد تجزیه و تحلیل قرار گرفت. یافته‌ها: آزمون آماری کای دو نشان داد دو گروه از نظر متغیرهای دموگرافیک مثل سن، درآمد و سطح تحصیلات و... یکسان هستند (P>0/05). همچنین تی زوجی در گروه آزمون نیز اختلاف معناداری را قبل و بعد از مداخله در بین ابعاد کیفیت زندگی نشان داد (P<0/05)، لیکن در گروه شاهد این اختلاف معنادار نبود (P<0/05). نتیجه‌گیری: نتایج پژوهش نشان داد که اجرای برنامه حمایتی سلامت‌محور بر کیفیت زندگی مادران کودکان فلج مغزی مؤثر می‌باشد، لذا پیشنهاد می‌گردد برنامه حمایتی سلامت‌محور بعنوان یک روش مؤثر در ارتقاء کیفیت زندگی مادران کودکان با بیماری‌های مزمن بکار‌گرفته شود.

Title: گزارش یک بیمار فارسی زبان راست دست با زبان پریشی متقاطع (CAD ): شواهدی از علائم دستور پریشی در اثر آسیب نیمکره راست

http://rehabilitationj.uswr.ac.ir/article-1-810-fa.html

زبان‌پریشی متقاطع پدیده‌ای است که در آن فردی که دارای آسیب نیمکره راست مغز می‌باشد ، علائم زبان‌پریشی را نشان می‌دهد. این پدیده در دنیا نادر می‌باشد و تاکنون در زبان فارسی هیچ بیمار زبان‌پریش متقاطعی گزارش نشده است، هدف از این مقاله آن است که گزارش کاملی از یک بیمارزبان‌پریش 31 ساله فارسی زبان با آسیب نیمکره راست ارائه شود. در این مطالعه موردی از آزمون زبان‌پریشی دو زبانه (BAT)، آزمون زبان‌پریشی نامیدن و آزمون کنش‌پریشی برای ارزیابی بیمار استفاده شد، علاوه برآن بیش از 100 گفته از نمونه گفتار آزاد بیمار مورد تحلیل قرارگرفت. بیمار در اجرای آزمون نامیدن و کنش‌پریشی موفق بود ولی در آزمون زبان‌پریشی دوزبانه بیشترین مشکل را در قسمت ساخت جمله که احتیاج به کاربرد صحیح قواعد دستور زبان فارسی دارد، نشان دادو در قسمت درک نحوی، قضاوت دستوری، قضاوت واژگانی، روانی کلامی ودرک خواندن هم نقص نشان داد. تحلیل زبانشناختی نمونه گفتار بیمار علاوه بر سرعت پایین گفتار وی، نقص در کاربرد حروف اضافه، خطا در کاربرد صحیح کلمات مرکب و ترکیبات فعلی را نیز نشان داد. بر اساس یافته‌های پژوهش ضمن تأییدزبان‌پریش بودن بیمار، شواهدی از نقص در پیوستگی و انسجام گفتار پیوسته نیز مشاهده شد که از یک‌سو می‌تواند به دلیل ناتوانی در کاربرد صحیح جملات پیچیده و عدم استفاده صحیح از نشانگرهای گفتمان (نقص در کاربرد قواعد دستوری) باشد و از سوی دیگر به دلیل عدم حفظ موضوع، حاشیه پردازی، نقص در بیان روشن مراجع ضمایر و مشکل در انتخاب واژگان صحیح برای بیان منظور (یعنی نقایص کاربرد شناسی زبان که در اثر آسیب نیمکره راست ایجاد می‌شود) باشد. در مجموع اینطور به نظر می‌رسد که بیمار مورد نظر ترکیبی از علائم زبان‌پریشی ناروان، دستور‌پریشی و علائم نیمکره راست را نشان می‌دهد.

Title: مقایسه اثربخشی روش تحلیل رفتار کاربردی و روش درمانی-آموزشی بر رفتار کلیشه‌ای، مشکلات تعاملی و ارتباطی کودکان مبتلا به اوتیسم

http://rehabilitationj.uswr.ac.ir/article-1-794-fa.html

هدف: ‌این پژوهش با هدف مقایسه اثربخشی روش تحلیل رفتار کاربردی و روش درمانی-آموزشی بر رفتار کلیشه‌ای، مشکلات تعاملی و ارتباطی کودکان مبتلا به اوتیسم انجام گرفت. روش بررسی:‌ در این مطالعه آزمایشی، جامعه آماری شامل کلیه کودکان مدرسه اوتیسم تبریز در شش ماهه دوم سال 1388 می‌باشد حجم نمونه شامل 29 کودک (21 پسر و 8 دختر) بود که در دامنه سنی 6 تا 14 سال قرار داشتند و با روش نمونه گیری تصادفی انتخاب و در گروه تحلیل رفتار کاربردی (8 پسر و 2 دختر) گروه درمانی- آموزشی (9 پسر و 1 دختر) و گواه (4 پسر و 5 دختر) جایگزین شدند. ابزارهای این پژوهش عبارت بودند از: چک لیست بازبینی اصلاح شده و مقیاس درجه بندی اوتیسم گیلیام. داده‌های بدست آمده با استفاده از آزمون آماری کوواریانس تحلیل گردید. یافته‌ها: نتایج این پژوهش نشان داد که میانگین شاخص‌های مشکلات رفتاری گروههای آزمایشی روش تحلیل رفتار کاربردی و روش درمانی- آموزشی در مقایسه با گروه گواه به طور معناداری کاهش پیدا کرده است (۰/۰۱>P). همچنین در مقایسه دو روش درمانی تحلیل رفتار کاربردی و درمانی-آموزشی کاهش میانگین نمرات در شاخص مشکلات ارتباطی به نفع درمان تحلیل رفتار کاربردی و معنادار می‌باشد (۰/۰۵>P) نتیجه‌گیری: با توجه به یافته‌های پژوهش، هر دو روش درمانی تحلیل رفتار کاربردی و درمانی- آموزشی در کاهش نشانه‌های مشکلات رفتاری کودکان مبتلا به اوتیسم، مؤثر بوده‌اند. با این وجود، درمان تحلیل رفتار کاربردی، به دلیل اثربخشی بیشتر، درمان پیشنهادی این پژوهش می‌باشد.

Title: بررسی تأثیر ماساژ SSBM بر درد شانه بیماران مبتلا به سکته مغزی مراجعه‌کننده به مرکز توان‌بخشی تبسم شهر تهران در سال 1389

http://rehabilitationj.uswr.ac.ir/article-1-800-fa.html

هدف: درد شانه ناشی از همی‌پلژی یک یافته شایع پس از سکته ‌مغزی می‌باشد که استقلال بیمار در انجام فعالیتهای روزمره به شدت محدود می‌نماید پژوهش حاضر به منظور تعیین تأثیر ماساژ اس‌اس‌بی‌ام بر درد‌شانه بیماران مبتلا به سکته مغزی انجام شده است. روش بررسی: پژوهش حاضر یک مطالعه نیمه تجربی است، شرکت کنندگان این مطالعه شامل 34 بیمار واجد معیارهای ورود بودند که از میان 70 بیمار مراجعه کننده به مرکز توانبخشی تبسم در فاصله زمانی ۱۳۸۹/۸/۱ تا ۱۳۸۹/۱۰/۱ انتخاب شدند و به روش تصادفی در دو گروه مداخله و شاهد قرار گرفتند. برای هر یک از نمونه‌های گروه مداخله 7 جلسه متوالیروزانه ماساژ اس‌اس‌بی‌ام به مدت 10 دقیقه انجام شد. ابزار گرد‌آوری داده‌ها شاملپرسشنامه مشخصات دموگرافیک، مقیاس دیداری درد و بود. میزان درد شانه قبل و 24 ساعت پس از آخرین جلسه ماساژ در هر دو گروه مداخله و شاهد ارزیابی شد و همچنین بین دو گروه مورد مقایسه قرار گرفت. تجزیه و تحلیل داده‌ها توسط آزمونهای آماری توصیفی و تحلیلی (تی مستقل، تی زوجی، آنالیز کوواریانس) صورت گرفت. یافته‌ها: میانگین درد شانه قبل از مداخله درگروه آزمون ۸/۴۰ و در گروه شاهد ۸/۴۰ پس از مداخله میانگین درد شانهدر گروه مداخله ۱/۱۳ و در گروه شاهد ۸/۴۰ محاسبه گردید. نتایج در سطح %95 از لحاظ آماری معنا دار بود. نتیجه‌گیری: با توجه به نتایج حاصل از پژوهش حاضر، اموزش و بکارگیری ماساژ اس‌اس‌بی‌ام توسط پرسنل درمانی و مراقبتی می‌تواند گام مؤثری در پیشگیری یا کاهش درد شانه و بالطبع بهبود عملکرد حرکتی بیماران مبتلا به همی‌پلژی در اثر سکته مغزی محسوب گردد.

Title: بررسی پایایی اولتراسونوگرافی توان‌بخشی و ارتباط بین اندازه‌گیری خطی و اندازه‌گیری سطح مقطع عضلات مولتی فیدوس کمری در افراد سالم

http://rehabilitationj.uswr.ac.ir/article-1-914-fa.html

هدف: استفاده از اولتراسونوگرافی توانبخشی امروزه در کلینیک‌های فیزیوتراپی کشورهای پیشرفته در حال گسترش است. به همین لحاظ توجه به تکرارپذیری تصاویر سونوگرافی بدست آمده توسط فیزیوتراپیستها و همینطور روش‌های مورد استفاده در ارزیابی اندازه عضلات از اهمیت خاصی برخوردار است. این مطالعه به منظور ارزیابی تکرارپذیری و ارتباط بین دو روش اندازه‌گیری سطح مقطع و روش خطی برای تعیین اندازه عضلات مولتی فیدوس کمری با استفاده از تصاویر سونوگرافی تهیه شده توسط فیزیوتراپیست تازه آموزش دیده طراحی شده است. روش بررسی: تصویر سونوگرافی عضلات مولتی فیدوس دو طرفه سطوح L2 تا L5 کمری 60 داوطلب خانم سالم ثبت شد. سپس سطح مقطع عضلات مولتی فیدوس با دو روش 1) رسم حاشیه عضله و محاسبه سطح درون حاشیه رسم شده، و 2) روش خطی رسم قطرهای طرفی و قدامی-خلفی و ضرب این قطرها در یکدیگر محاسبه شد. تکرارپذیری داده‌ها نیز در یک مطالعه موازی با حضور 16 داوطلب سالم و اندازه‌گیری‌های فوق در دو جلسه متوالی بررسی گردید. یافته‌ها: پایایی معناداری بین دو جلسه ارزیابی برای هر دو روش اندازه‌گیری خطی و سطح مقطع در تمام سطوح مهره‌های کمری مشاهده گردید (ICC=0/81-0/98). ارتباط معناداری بین دو روش سطح مقطع و خطی برای تعیین اندازه عضلات مولتی فیدوس کمری یافت شد (r=0/89-0/97, P<0/0001). نتیجه‌گیری: این یافته‌ها نشان داد که استفاده از سونوگرافی توانبخشی و روش‌های ارزیابی خطی در کلینیک‌های فیزیوتراپی روش مؤثری برای ارزیابی اندازه و تغییرات عضله مولتی فیدوس کمری در برنامه‌های توانبخشی است.

Title: بررسی عوامل مؤثر بر استفاده از نتایج و شواهد پژوهشی در فعالیت‌های بالینی کارشناسان توان‌بخشی بیمارستان‌های آموزشی شهر تهران

http://rehabilitationj.uswr.ac.ir/article-1-973-fa.html

هدف: این پژوهش با هدف تعیین عوامل مؤثر بر استفاده از نتایج و شواهد پژوهشی در فعالیت‌های بالینی کارشناسان توانبخشی بیمارستان‌های آموزشی شهر تهران، انجام شده است. روش بررسی: جامعه آماری پژوهش، کارشناسان توانبخشی بیمارستان‌های آموزشی شهر تهران (485 نفر) بودند. ابزار گردآوری داده‌ها، پرسشنامه‌های تعیین موانع و تسهیل کننده‌های استفاده از شواهد پژوهشی فانک بود. در نمونه گیری به روش تصادفی خوشه‌ای، 165 نفر از کارشناسان انتخاب شدند. از آمار توصیفی و آزمون‌های تی مستقل و آنالیز واریانس یکطرفه جهت تحلیل داده‌ها استفاده شد. یافته‌ها: ٪۷۰/۵ شرکت کنندگان در پژوهش، زنان با میانگین سنی 38 و سابقه کاری 12 سال بودند. اغلب آنها (٪۷۸/۳) دارای مدرک لیسانس و فیزیوتراپیست بودند. شرکت کنندگان در اختیار نداشتن تسهیلات کافی جهت بکارگیری یافته‌های تحقیق و عدم برخورداری ازحمایت قانونی جهت تغییر در روش‌های انجام کار را به عنوان مهم ترین موانع و افزایش انگیزه فردی در بکارگیری نتایج تحقیقات و فراهم آوردن امکاناتی از قبیل کامپیوتر و دسترسی به اینترنت را به عنوان مهم ترین تسهیل کننده‌های عملکرد مبتنی بر شواهد برشمردند. نتیجه‌گیری: کارشناسان توانبخشی، مهم ترین موانع و تسهیل کننده‌های استفاده از شواهد پژوهشی را فراهم نبودن تسهیلات کافی برای بکارگیری نتایج تحقیق و افزایش علاقمندی و انگیزه فردی در به عمل آوردن شواهد پژوهشی دانستند.

Title: بررسی تأثیر تمرینات توان‌بخشی بر همبستگی الگوهای زمانی در راه‌رفتن افراد با ضایعه لیگامان متقاطع قدامی

http://rehabilitationj.uswr.ac.ir/article-1-934-fa.html

هدف: در این تحقیق اثر تمرینات اغتشاشی بر روی شاخص همبستگی آنالیز افت و خیز بدون روند (&alpha) برای سیکلهای راه رفتن در افراد مبتلا به ضایعه لیگامان صلیبی قدامی، با روش‌های محاسباتی دینامیک غیرخطی بررسی شده است. روش بررسی: مطالعه بر روی 10 مرد ورزشکار مبتلا به ضایعه لیگامان صلیبی قدامی که حداقل 6 ماه از ضایعه لیگامانی آنها گذشته بود صورت گرفت. آزمودنی‌ها به روش غیراحتمالی انتخاب شدند. با استفاده از داده⁭های سوییچ⁭های پایی، زمان گام برای حداقل 270 سیکل راه رفتن برای هر فرد قبل و بعد از اغتشاش درمانی استخراج شد و جهت آنالیز شاخص همبستگی آنالیز افت و خیز بدون روند (&alpha) مورد استفاده قرار گرفت. یافته⁭ها: نتایج نشان دهنده تفاوت مقدار &alpha برای داده⁭های اصلی و در هم ریخته شده است که نشانگر اتفاقی نبودن ساختار اولیه داده⁭های اصلی است. با توجه به قرار گرفتن &alpha در محدوده (0/5>&alpha>1) همبستگی بلند مدت بین سیکل⁭های راه رفتن دیده شد. همچنین نتایج بیانگر عدم تفاوت معنی دار &alpha قبل و بعد از درمان می⁭باشد (۰/۵۱۵=P). نتیجه⁭گیری: نتایج مربوط به شاخص همبستگی، نشان دهنده وجود وابستگی الگوهای زمانی می⁭باشد که به راه رفتن با سرعت آرام نزدیک است. همچنین می⁭توان نتیجه گرفت که از لحاظ الگوهای همبستگی زمانی افراد مبتلا به ضایعه لیگامان صلیبی قدامی رفتاری مشابه با افراد سالم دارند.

Title: گزارش موردی تأثیر تزریق بوتولینوم توکسین بر عملکرد اندام فوقانی سمت مبتلای یک بیمار پس از گذشت 16 سال از سکته مغزی

http://rehabilitationj.uswr.ac.ir/article-1-641-fa.html

هدف: تزریق بوتولینوم توکسین یکی از روش‌های کاهش اسپاستیسیته و در نتیجه برگرداندن عملکرد اندام فوقانی در افراد سکته مغزی می‌باشد. هدف از این مطالعه موردی، گزارش تأثیر تزریق بوتولینوم توکسین بر روی عملکرد اندام فوقانی بعد از گذشت 16 سال از سکته‌ مغزی می‌باشد. روش بررسی: بیمار مردی 63 ساله بود که 16 سال پیش دچار سکته مغزی در نیمکره چپ مغز و در نتیجه‌ آن پارزی در دست و بازوی غالب شده است البته بیمار قادر به انجام حرکات ساده در سمت مبتلا بود اما از کندی و سفتی حرکات شکایت داشت. برای درمان اسپاستیسیته از داروی بوتولینوم توکسین نوع A استفاده شد. تزریق در عضلات انتخاب شده اندام فوقانی مبتلا توسط پزشک متخصص و با دوزی خاص براساس حجم هر عضله بین 150-50 واحد انجام شد. بعد از تزریق دارو بیمار به مدت 3 ماه تحت نظر بود و در پایان هر ماه ارزیابی‌های مورد نظر انجام می‌گرفت. با وجود پیشنهاد تیم تحقیقاتی مبنی بر ادامه درمان توانبخشی بیمار بدلایل شخصی در این مدت در هیچ برنامه درمانی شرکت نکرد و ضمنا داروی ضد اسپاستیسیته دیگری نیز دریافت نمیکرد. یافته‌ها: دامنه‌ حرکتی تمام مفاصل بهبود داشت ولی این بهبودی چشمگیر نبود. بیشترین بهبودی در دامنه‌ حرکتی اکتیو و پاسیو مفاصل متاکارپوفالنژیال دیده شد. در عملکرد اندام فوقانی به میزان قابل توجهی در پایان هر ماه نسبت به شروع درمان پیشرفت حاصل شد. بیشترین بهبودی عملکرد اندام فوقانی مربوط به قسمت دست مقیاس فوگل-میر بود. در پایان ماه اول اسپاستیسیته به میزان قابل توجهی کاهش یافت به طوریکه بیمار با تلاش کمتری نسبت به قبل از تزریق، اکستنشن را در همه مفاصل انجام می‌داد. هر چند که اسپاستیسیته هیچ گاه به حد صفر نرسید. نتیجه‌گیری: تزریق بوتولینوم توکسین بعد از گذشت 16 سال از سکته مغزی نیز ممکن است باعث بهبود عملکرد دست شود.

Title: کم‌وزنی هنگام تولد و عوامل مرتبط با آن درایران: برطبق مدل سازمان جهانی بهداشت

http://rehabilitationj.uswr.ac.ir/article-1-933-fa.html

هدف: وزن هنگام تولد یکی از مهمترین ،ساده ترین ور ایج ترین شاخص های بهداشتی،برای ارزیابی وضعیت نوزادان درهر کشور است.این پژوهش به منظور بررسی مقالات انجام شده در ارتباط با شیوع و عوامل مرتبط با کم وزنی نوزادان درایران انجام شده است. روش بررسی: این مطالعه به مرور ، تحقیقاتی می پردازد که شیوع و عوامل مرتبط با کم وزنی نوزادان را درهنگام تولد ، در 10 سال اخیر،مورد بررسی قرارداده اند.مقالات باجستجوی اطلاعات موجوددر ۶ پایگاه اطاعاتی(پاپ مد، ایران مدکس، اس آی دی ، ایران داک، اسکولار گوگل، مگ ایران) ،منابع موجود درهر مقاله، وبا استفاده ازکلمات کلیدی،کم وزنی،مرگ ومیر نوزادان، عوامل مرتبط با کم وزنی، شیوع، عوامل اجتماعی موثر بر سلامت، مورد بررسی قرار گرفتند. یافته‌ها: در کل از36 مطالعه مورد بررسی 14 مقاله عوامل مرتبط با کم وزنی و 22 مقاله هم شیوع و هم عوامل مرتبط با کم وزنی را در هنگام تولد گزارش نمودند که در بیش از %70 آنها شیوع کم وزنی ،(%12-5)و در5 مورد شیوع آن (%13-1/19) گزارش شده است. عوامل مرتبط با کم وزنی هنگام تولد در مقالات عبارت بودند از عوامل دموگرافیک ،عوامل اقتصادی اجتماعی، سبک زندگی وحمایت اجتماعی که دراین بین عوامل دموگرافیک بیشترین ارتباط را داشتند. نتیجه‌گیری: گرچه تفاوت هایی (از جمله تفاوت در تعریف کم وزنی ، کم یا عدم گزارش دهی ، کم بودن حجم نمونه و...) در روش مطالعات وجود داشته است، اما بطورکلی شیوع کم وزنی در ایران نسبتا بالا بوده لذا آموزش، افزایش مراقبت های دوران بارداری بویژه برای گروه سنی پرخطر را برای کاهش این پیامد بد بارداری پیشنهاد می نماییم

Title: بررسی حافظه کلامی و دیداری بیماران با تشخیص اسکیزوفرنیا با توجه به سیر بیماری و سطح هوش عمومی

http://rehabilitationj.uswr.ac.ir/article-1-462-fa.html

هدف: ارزیابی عملکردهای شناختی در بیمار اسکیزوفرنیا از اهمیت بالایی برخوردار است، چرا که نقایص شناختی در حافظه و هوش از جنبه های اساسی و زیربنایی بوده، وهر دو از یک نقص ساختاری و عصب شناختی مشابه ای حکایت می‌کنند. پژوهش حاضر به دنبال بررسی تاثیر شدت وسیر اختلال اسکیزوفرنیا بر عملکرد شناختی حافظه و هوش و همچنین رابطه هوش و حافظه با یکدیگر است. روش بررسی: در این پژوهش 60 بیمار که حداقل دارای 2 سال سابقه تشخیص اسکیزوفرنیا بودند، بر اساس مصاحبه به وسیله مقیاس PANSS در دو گروه 30 نفره شامل گروه اول با علائم خفیف (گروه باثبات ) و گروه دوم با علائم شدید (روبه زوال)، مورد مطالعه قرار گرفتند. دو گروه از طریق سن، سواد، عدم تشخیص عقب ماندگی ذهنی قبل از بیماری، عدم استفاده از الکتروشوک، عدم قطع درمان دارویی ضد روان‌پریشی بیشتر از سه ماه طی یک سال گذشته مورد همتاسازی قرار گرفتند، با آزمون حافظه و کسلر و آزمون هوش ریون بزرگسالان مورد آزمون قرار گرفتند و نتایج با آزمون‌های آماری تی تست مستقل، ضریب همبستگی و رگرسیون مورد بررسی قرارگرفت. یافته‌ها: بین میانگین بهره‌ی حافظه‌ی گروه باثبات با 77/4 و گروه رو به زوال با 65/93 تفاوت معنی دار (P=0/002) وجود داشت. ضمناً تفاوت میانگین هوش عمومی گروه باثبات با 84/26 و گروه رو به زوال با 76/9 از لحاظ آماری معنی داری (P=/015) بود. آزمون رگرسیون نشان داد که از طریق بهره هوشی می‌توان حافظه را پیش بینی کرد (P=0/001). نتیجه‌گیری: با شدت پیداکردن بیماری اسکیزوفرنیا و سیر رو به زوال و وجود علائم منفی، نقص در حافظه افزایش یافته است. همچنین در این بیماران، بین هوش و حافظه رابطه مثبت وجود داشت. سطح هوش گروه رو به زوال به طور معنی داری پایین‌تر از گروه با علائم کم و با ثبات بود.
